# Unveiling the role of long non-coding RNA MALAT1: a comprehensive review on myocardial infarction

**DOI:** 10.3389/fcvm.2024.1429858

**Published:** 2024-08-07

**Authors:** Reza Eshraghi, Sina Sadati, Ashkan Bahrami, Seyed Reza Mirjalili, Alireza Farrokhian, Maryam Mahjoubin-Tehran, Hamed Mirzaei

**Affiliations:** ^1^ Student Research Committee, Kashan University of Medical Sciences, Kashan, Iran; ^2^ Yazd Cardiovascular Research Center, Non-Communicable Diseases Research Institute, Shahid Sadoughi University of Medical Sciences, Yazd, Iran; ^3^ Department of Cardiology, School of Medicine, Kashan University of Medical Sciences, Kashan, Iran; ^4^ Biotechnology Research Center, Pharmaceutical Technology Institute, Mashhad University of Medical Sciences, Mashhad, Iran; ^5^ Research Center for Biochemistry and Nutrition in Metabolic Diseases, Institute for Basic Sciences, Kashan University of Medical Sciences, Kashan, Iran

**Keywords:** myocardial infarction, long non-coding RNA, metastasis associated lung adenocarcinoma transcript 1, epigenetic, pathology

## Abstract

Myocardial infarction (MI) stands at top global causes of death in developed countries, owing mostly to atherosclerotic plaque growth and endothelial injury-induced reduction in coronary blood flow. While early reperfusion techniques have improved outcomes, long-term treatment continues to be difficult. The function of lncRNAs extends to regulating gene expression in various conditions, both physiological and pathological, such as cardiovascular diseases. The objective of this research is to extensively evaluate the significance of the lncRNA called Metastasis associated lung adenocarcinoma transcript 1 (MALAT1) in the development and management of MI. According to research, MALAT1 is implicated in processes such as autophagy, apoptosis, cell proliferation, and inflammation in the cardiovascular system. This investigation examines recent research examining the effects of MALAT1 on heart function and its potential as a mean of diagnosis and treatment for post- MI complications and ischemic reperfusion injury.

## 1 Introduction

A considerable reduction in the blood flow to the coronary arteries causes an abrupt heart attack, known as acute MI (AMI), which is considered the foremost cause of mortality in industrialized countries. The main behind-the-scene mechanisms are atherosclerotic plaque formation and endothelial injury lipid accumulation (1). In this situation, plaque rupture by forming thrombosis restrains blood flow. Nonetheless, coronary artery embolism, coronary dissection, cocaine abuse, and coronary vasospasm are other non-common causes of AMI (2, 3). The duration and the magnitude of occlusion- the reduction rate of blood flow- significantly affect the post-MI myocardial function; hence, rapid reperfusion is vital. Beyond the rapid reperfusion managements including percutaneous coronary intervention (PCI), fibrinolysis, and surgery, the long-term approach is fundamentally focused on bicellular supports including lipid-lowering therapy, antithrombotic therapy, and β-blocker treatment (4). However, in spite of all recent progresses in multiple novel therapies, we could not lessen the complications and morbidities (5). In this regard, besides preventing interventions like improving life style, we need to develop new strategies to decrease the post-infract conditions.

Long non-coding RNAs (lncRNAs) are a type of transcripts that are made up of more than 200 nucleotides (6). LncRNAs play a crucial role in the regulation of gene expression. This is achieved through their influence on mRNA stability, along with their involvement in post-translational modification. Additionally, lncRNAs also function as competitive endogenous RNAs, further contributing to their regulatory capabilities (7). Considering the importance of proteins in various diseases, a considerable number of investigations on lncRNAs have been performed that have demonstrated associations between lncRNAs and prostate cancer, gastric cancer, lung cancer, and cardiovascular diseases (8–10). Moreover, different studies showed the lncRNAs influences on not only development, but also progression of MI, and indicated diagnostic or therapeutic potentials of lncRNAs in MI (11–13).

One of the most thoroughly researched lncRNAs in the field is called MALAT1. This particular lncRNA has been primarily found in relation to lung metastasis and is used as a means of predicting prognosis (14). Later, its profound expression in endothelial cells (ECs) and cardiomyocytes experiencing high glucose, hypoxic situation, and oxidative stress drew the attentions to the MALAT1 potentials in MI management (15–18). Therefore, in this comprehensive review, we compile the forefront findings pertaining to the functions and underlying mechanistic insights into the MALAT1 in the context of MI.

## 2 MALAT1 and MI

**Figure 1:**
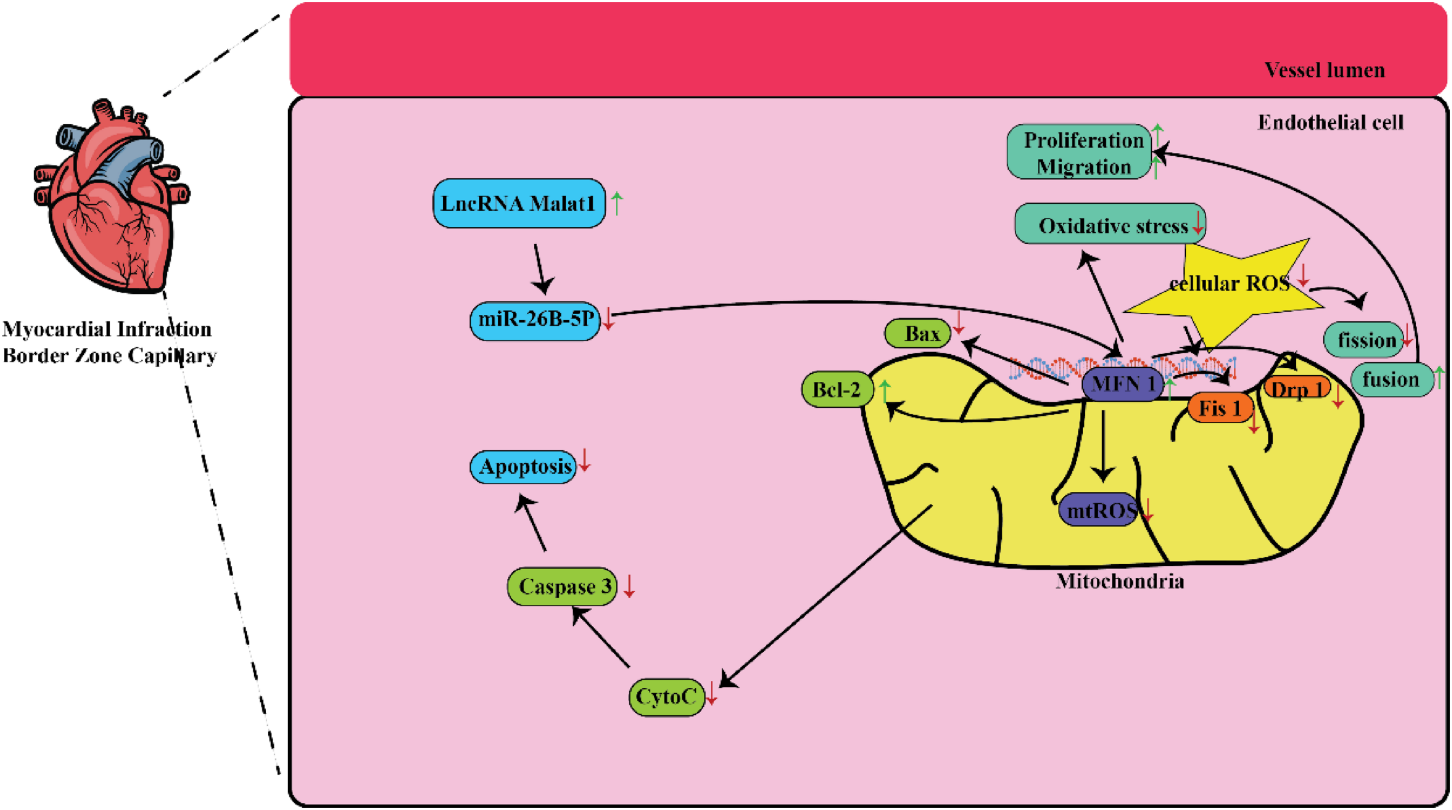
Some mechanisms that are targeted by MALAT1 in MI. MALAT1-mediated mitochondrial homeostasis enhanced cardiac microcirculation resistance after MI via the miR-26b-5p/Mfn1 axis.

In a study conducted in a medical setting, it has been found that the lncRNA referred to as lnc-MALAT1 is linked to a higher likelihood of developing coronary artery disease (CAD) and a larger size of stenosis in the coronary arteries (19, 20). The initial stage of healing a heart injury begins with the inflammatory process that occurs after an acute MI. The procedure involves activating the body's natural defense mechanism and drawing in infection-fighting white blood cells to remove deceased cells from the site of injury. This process requires the participation of reactive oxygen species, the complement system, and the activation of various chemokines (21). Furthermore, a multitude of different cells, such as lymphocytes, macrophages, other types of immune cells, fibroblasts, and endothelial cells, become activated and have a part in the progression of healing (22). Figure 1 shows some mechanisms that can be targeted by MALAT 1 in MI. Table 1 lists different studies on MALAT1 and MI.

**Table 1:** Studies on the role of MALAT1 in MI.

Disease model	Model	MI model method:	Expression in disease	Sample ex: PBS, heart tissue	Implication	Mechanism and target	Ref
MI	*In vitro* (NRCMs)	*In vitro*: hypoxic condition which consisted of 94% N_2_, 5% CO_2_, and 1% O_2_	Increased		Induced autophagy to block hypoxia-induced cell injury	ULK1	(23)
MI	*In vivo*/*In vitro*	*In vivo*: LAD ligation	Knockdown	Heart tissue	MALAT1 knockdown inhibited proliferation and reduced scar size 1 week and 3 weeks after MI	hnRNP U	(24)
MI	*In vivo* (617 patients)	–	Not evaluated	Peripheral venous blood samples	The MALAT1 rs3200401 CT + TT genotypes could be a risk factor for MACCEs in MI patients	–	(25)
MI receiving PCI	*In vivo* (198 STEMI patient)	–	Increased	Serum	MALAT1 functioned effectively as a biomarker of no-reflow phenomenon in STEMI patients receiving pPCI.	miR-30e, miR-155, miR-126, CRP, EDN1, and HPSE.	(26)
MI	*In vivo* (male C57BL/6J mice)/*in vitro* (CMECs)	*In vivo*: LAD ligation	Inhibited	serum and cardiac tissues	Malat1 knockdown severely exacerbated cardiac remodeling and dysfunction, and increased fibrosis deposition area. It also regulated angiogenesis and endothelium-derived vasodilatation after MI	miR-26b-5p/Mfn1, Mcl-1, Bax, caspase 3 Bcl-2,, and Bcl-xL w	(27)
MI	*In vivo* (C57BL/6J mice)/*in vitro*	*In vivo*: LAD ligation	Increased	myocardial tissue	Inhibited angiogenesis and myocardial regeneration	miR-25-3p, CDC42, MEK/ERK pathway	(28)
MI	*In vitro* (H9c2)	H9c2 cells were cultured in a hypoxic incubator with 3% O_2_ concentration for 24 h	Increased	–	Alleviated hypoxia induced H9c2 cell injury, promoted cell migration, viability, and invasion but inhibited cell apoptosis	miR217, Sirt1, PI3K/AKT, and Notch pathways	(29)
MI	*In vitro* (H9C2)	OGD and Reoxygenation (OGD/R)	Increased	–	Restored OGD/R-induced H9C2 cell Autophagy	miR-20b and beclin1	(30)
MI	*In vivo* (Male C57BL/6 mice)/*in vitro*	LAD ligation	Increased	Heart tissue	Promoted fibroblast proliferation, collagen exacerbated AngII-induced cell proliferation, and myofibroblast trans differentiation	TGF-β1 and miR-145	(31)
MI	*In vivo* (male Sprague Dawley)/*in vitro*	*In vivo*: LAD ligation	Induced and suppressed	heart tissues and PBS	Increased apoptosis and inflammatory factors IL-8, IL-1β, and IL-6	Caspase3, Bcl-2, MAPK, and ERK2	(32)
MI	*In vivo* (male Wistar rats)/*In vitro* (H9C2)	*In vitro* and *in vivo*: ISO-induced MI	Increased	–	Protected cardiomyocytes from apoptosis, decreased mitochondrial ROS levels, and conferred protective influences against MI via promoting autophagy and decreased apoptosis	miR-558, ULK1 mRNA, and Atg1	(33)
AMI	*In vivo* (72 male C57BL)/*In vitro*	*In vivo*: LAD ligation without chest opening and ventilation	Increased	Heart tissue	Deteriorated collagen deposition and inflammation	EZH2 and H3K27	(34)
AMI	*In vivo* (132 patientss with AMI)	Patients	Increased	blood sample	MALAT1 might be practical diagnostic biomarker for AMI and was negatively associated with NYHA and genisini score	–	(35)
AMI	*In vivo* (60 patients)/*in vitro* (HUVECs)	–	Increased	Blood samples	Regulated cell proliferation, apoptosis, migration, invasion, and angiogenesis. MALAT1 was shown to be potential novel biomarkers for diagnosis	BCL-2, BAX, caspase-3, PI3K/AKT	(36)
AMI	*In vivo* (160 patient)	–	Increased	Blood samples (PBMC)	lnc-MALAT1 increased atherosclerosis, inflammation, and myocardial damage, which directly increased the risk of MACE	nuclear factor-kappa B and Wnt/β-catenin	(37)
AMI	*In vivo* (32 adult male Sprague-Dawley rats)/*in vitro* (HL-1)	*In vivo*: LAD ligation and reperfusion by cutting the knot in the ligature, 30 min after ligation.	knockdown	Heart tissue	Induced apoptosis	miR-125b-5p, NLRC5	(38)
AMI	*In vivo* (Wistar male rats)/*in vitro*	LAD ligation	Increased after 1 day and decreased at 14 and 28 says post-MI.	Left ventricular tissue	Increased angiogenesis	miR-92a, KLF2, and CD31	(39)
STEMI	*In vivo* (50 patients)/*in vitro* (HUVECs)	–	Increased	Plasma	Promoted cell proliferation and migration of stem cells in hypoxic conditions and regulated intermittent hypoxia-induced injury of HUVECs. MALAT1 independently predicted MACE	miR-142-3p and miR-155-5p	(40)
Hypoxia model	*In vitro* (CPCs)	*In vitro*: applied CoCl2	Induced/suppressed	–	Enhanced the proliferation and migration potentials of CPCs under hypoxic conditions	JMJD6 and miR-125.	(41)
MIRI	*In vivo*/*in vitro* (H9c2 and HL-1)	*In vivo*: LAD ligation/*in vitro*: cells were treated by hypoxia/reoxygenation (HR)	Increased	Myocardial tissues	Expressed a pro apoptotic effect in myocardial injury	miR-133a-3p, IGF1R, PI3K/Akt/eNOS	(42)
MIRI	*In vivo* (120 Sprague-Dawley rats)/*In vitro* (H9c2 cells)	LAD ligation and after 40 min the ligature was loosened	Increased	Neonatal rat cardiomyocytes	Reduced the cell viability, increased apoptosis, and enhanced the autophagy	MiR-206 and ATG3.	(43)
MIRI	*In vivo* (12 male Sprague-Dawley rats)/*in vitro* (H9c2 cells)	LAD ligation and removal 30 min after ischemia and reperfusion was allowed for 60 min.	Increased	Cardiac tissue	Increased cell autophagy level and reduced cell apoptosis	miR-30a and BECN1	(44)
MIRI	*In vivo* (40 patients and male mice)/*in vitro* (HL-1)	Animal model: LAD ligation for 30 min and removal	Increased	Mice heart tissue and human PBS	Enhanced the H/R-induced cell apoptosis. MALAT1 showed diagnostic potentials	miR-144-3p,	(45)
MIRI	*In vivo* (12 male Sprague-Dawley rats)/*in vitro*	LAD ligation for 30 min and removal for 24 h.	Increased	Heart tissue	Increased myocardial apoptosis	miR-320 and Pten	(46)
MIRI	*In vivo* (Male C57BL/6 mice)/*in vitro* (HL-1)	LAD ligation for 45 min and removal	Increased	Heart tissue	Increased LDH release and cell apoptosis	miR-145 and Bnip3	(47)

### 2.1 MALAT1 and angiogenesis in MI

Visualize the creation of novel blood vessels from preexisting ones as the intricate process of angiogenesis. This complex sequence commences with the generation of endothelial cells (ECs), which then bind together and forge bonds with the surrounding extracellular matrix (ECM) (48). ECs can be divided into three primary groups: tip, stalk, and phalanx cells. Those situated at the distal end of blood vessels, namely the stalk and tip cells, have the ability to produce various pro-angiogenic substances, such as platelet-derived growth factor, vascular endothelial growth factor (VEGF), and fibroblast growth factor (49, 50).

To treat ischemic heart disease, angiogenesis holds an essential position (51). On the other hand, particular research has shown a direct connection between angiogenesis and the onset of atherosclerosis, as well as a higher likelihood of plaque rupture (52). ECs can be prompted to angiogenesis in response to hypoxia, owing to the activation of VEGF signaling. In addition, hypoxia can induce the synthesis of MALAT1. Suppression of MALAT1 results in a notable drop in VEGF expression, which in turn reduces the capacity of ECs to generate fresh blood vessels (53–55). One way this procedure takes place is by inhibiting 15-lipoxygenase 1 and stimulating signal transducers and activators of transcription 3 via the addition of a phosphate group (56).

The miR-26 family is extensively researched specially, examined the function of miR-26b-5p in different disease processes such as cellular proliferation, angiogenesis, and immunity. As an example, it was discovered that miR-26b-5p is a molecule that inhibits cellular growth and enhances cellular apoptosis in cases of liver cancer (57). Specifically by inhibiting the PDGF receptor-β, miR-26b-5p effectively obstructed the generation of fresh blood vessels and restricted the production of fibers in the livers of mice (58).. The process of stopping angiogenesis is achieved by reducing the concentrations of MMP2, snail, and VE-cadherin (57). The pathway consisting of lncMalat1, miR-26b-5p, and ULK2 that has an important effect in regulating autophagy and promoting cell survival in brain microvascular endothelial cells during episodes of oxygen-glucose deprivation and the subsequent recovery period (59). A screening process for miRNAs based on physical traits uncovers evidence that miR-26b-5p promotes the ability of ECs to survive and grow following a sudden shortage of oxygen (60).

In this context, Chen and colleagues investigated the extent of tissue injury in the cardiac muscles of mice and discovered a link between Malat1 and miR-26. This work demonstrated that the specifically reduced expression rate of Malat1 in ECs had a detrimental effect on oxidative stress, microvascular perfusion, angiogenesis, and ultimately cardiac function in MI models. Subsequently, suppressed Malat1 has been shown to significantly impede the growth, movement, and production of tubes in CMEC cells. This effect was partially caused by disrupted mitochondrial mediated apoptosis and dynamics. Moreover, through the utilization of bioinformatic investigations, luciferase assays, and pull-down assays, it was concluded that Malat1 operates as a competing endogenous RNA (ceRNA) for miR-26b-5p. Furthermore, it was found that Malat1employs Mfn1-mediated pathway to regulate endothelial function by means of modulating mitochondrial dynamics. Increased expression of Mfn1 significantly rectified the impaired function of small blood vessels and damage to CMEC cells that were worsened by suppressing Malat1, by inhibiting the excessive formation of fragmented mitochondria and apoptosis. They concluded that the Malat1 influences are likely due to its ability to inhibit the miR-26b-5p/Mfn1 pathway, which takes the responsibility for controlling mitochondrial dynamics and subsequent apoptosis (27).

The regulation of the MEK/ERK pathway, through therapeutic approaches such as insulin-like growth factor, statins, or post-ischemic conditioning, is widely acknowledged as a traditional method for treating myocardial ischemia injury (61). In contrast to MEK/ERK, the primary emphasis of studies on miR-25-3p have been conducted on its association with cancer. The presence of MiR-25-3p in human malignant cells stimulates angiogenesis via increasing the growth and movement of ECs. Consequently, this causes a raise in the movement and penetration of malignant cells (62). Chen et al. (28) the impact of M1-BMMs-EVs on the expression of CDC42 and the activation of the MEK/ERK pathway was thoroughly explored. This stimulation is a result of lncRNA MALAT1 being transferred and binding to miR-25-3p, resulting in a decrease in both angiogenesis and the ability of the heart to regenerate after a heart attack. MALAT1 was discovered to have increased levels in both MI mice, cells, and extracellular vehicles (EVs). MALAT1 and miR-25-3p were implemented in order to analyze the influence of extracellular vesicles on cells exposed to oxygen-glucose deprivation (OGD). In mice who experienced a MI, the administration of EV therapy had a negative impact on both the heart attack itself and the processes of angiogenesis and heart tissue regeneration. Utilizing EV treatment on cells subjected to oxygen-glucose deprivation causes a decline in the survival rate of cells, hindering their ability to reproduce and develop new blood vessels. The abundance of MALAT1 was notably amplified in MI mice, microvascular endothelial cells exposed to lack of oxygen and glucose, M1 macrophage derived from the bone marrow that displayed heightened inflammatory characteristics, and external vesicles released by these cells. Reducing the levels of MALAT1 decreased the ability of extracellular vesicle therapy to suppress the negative impact of oxygen-glucose deprivation on cells. The presence of MALAT1 acted as a sponge for miR-25-3p, leading to a rise in the expression of CDC42. Furthermore, increased levels of miR-25-3p resulted in enhanced cell survival, growth, and development of new blood vessels in cells subjected to oxygen-glucose deprivation. The use of EV therapy led to a significant rise in the functioning of the MEK/ERK pathway. The collaborative effect of M1-BMMs-derived EVs inhibited the formation of new blood vessels and hindered the repair of damaged heart tissue following a heart attack, through the influence of the MALAT1/miR-25-3p/CDC42 axis and the MEK/ERK pathway (28).

KLF-2 is a subgroup of zinc-finger transcription factors (63) that have significant involvement in vascular functioning (64) and are prominently presented in ECs (65). Shear stress induces the expression of KLF-2 (66), while certain lesions, comprising those resulted from proinflammatory substances or ischemic stroke, can block its expression (65, 67). KLF-2 functions as a transcription factor and specifically promotes S1P1 expression. On the other hand, KLF-4 controls several pathways, comprising the NF-κB pathway. An upregulation of Klf2 gene and protein expression in lung tissue and HLMVECs has been seen in response to HO, indicating a potential stress response (68). Interestingly, an elevation in KLF-2 is associated with a decrease in angiogenesis produced by HO.

To examine the changes of MALAT1 expression in myocardial generated exosomes in interaction with hyperbaric oxygenation (HBO), Shyu et al. have conducted an experimental study and put cultured cardiac myocytes in HBO at a pressure of 2.5 atmosphere. Exosomes originated from the growth medium. The researchers created an animal model to mimic acute MI by clamping the left anterior descending artery (LAD). HBO therapy significantly increased the amount of MALAT1 in heart muscle cells according to their findings. In addition, MALAT1 and exosomes induced by HBO significantly decreased the levels of miR-92a after MI. The expression rates of KLF2 and CD31 was markedly reduced following ligation, and the administration of HBO-induced exosomes effectively restored the expression. The expression rates of KLF2 and CD31 proteins were drastically reduced post-infarction using HBO-induced exosomes when MALAT1 was silenced using MALAT1-locked nucleic acid GapmeR. Exosomes generated by HBO therapy also considerably reduced the extent of the infarction. Exosomes derived from cardiac myocytes, stimulated by HBO, increased the expression of MALAT1. This led to the suppressed miR-92a expression and counteracted its inhibitory influence on CD31 and KLF2 expression in the myocardium of left ventricle (LV) after a heart attack. As a result, neovascularization was enhanced (39).

### 2.2 MALAT1 and apoptosis in MI

Programmed cell death occurs through the activation of distinct signaling pathways that ultimately result in the annihilation of cells (69). It is a crucial factor in different physiological processes, comprising homeostasis and the aging process (70–72). Apoptosis also significantly affects myocardial tissue following a sudden heart attack and leads to the LV remodeling and heart failure (73). Apoptosis holds a significant function in ischemic heart disease. It has a crucial activity in the death of myocytes during AMI and is mostly concentrated in the peri-infarcted zone (74, 75). During the middle stage of MI, a considerable number of cells die, which is believed to be linked to indications of ongoing changes to the structure of the left ventricle (76). Furthermore, patients, who experienced symptoms of heart failure just after AMI, exhibited a notable rise in apoptosis rates (77). Therefore, apoptosis is shown to have a substantial impact on acute MI.

In mouse models of cardiac ischemia/reperfusion (I/R), it was established that miR-320 acts as a prominent inducer of apoptosis. Results from experimental studies indicated that miR-320 facilitates programmed cell death in cardiac cells. In contrast, the suppression of miR-320 resulted in a decrease size in the infarcted area following I/R *in vivo* (78). This regulation is achieved by specifically targeting a protein called AKIP1 and activating the pathway that leads to cell death in the mitochondria (79).

In addition, emerging data indicates that the suppression of endogenous Pten leads to heart dysfunction and hypertrophy by deactivating the Pten-inducible kinase 1 (Pink1)/AMPK signaling pathway (80). PTEN is a genetic component that restrains tumor development and has the ability to impede the PI3K/Akt pathway, which is participating in the increase, endurance, and development of blood vessels (81). PTEN also controls the expression of other genes that involve in both the external and internal pathways of programmed cell death, including Bcl-2, Bid, Bax, caspases, and Sox-1 (82).

In this analysis, Hu and colleagues conducted a practical investigation on the modulatory function of miR-320 and Pten in the involvement of MALAT1 in acute MI (AMI). They employed qRT-PC and western blotting to evaluate the expression of these variables. The interrelationships between these factors were further confirmed by luciferase reporter examination. They demonstrated highly expression of MALAT1 and Pten but a least miR-320 expression in mice following AMI. In this relationship, MALAT1 sponges miR-320 to increase the expression of Pten. They concluded that suppressing MALAT1 expression can alleviate myocardial apoptosis via inhibiting Pten (46).

NOD-like receptors (NLRs) play various functions in the natural defense mechanisms within the body (83). The investigation of NLRC5 has largely concentrated on its function in controlling the display of antigens by altering the expression of major histocompatibility complex class I genes (84). NLRC5 is associated with blocking the production of type I interferons (85). Research has proven that NLRC5 involves substantially in the process of malignant cells changing and invading other tissues (86, 87). Furthermore, a growing body of studies has discovered a connection between NLRC5 and non-immunological disorders. MiR-125b-5p has been known as a well-established controller of apoptosis in several cell types (88–90). Furthermore, NLRC5 has recently been found to possess a cardioprotective impact against heart disease (91).

The survival and proliferation rates are influenced by the PI3K/Akt and Notch signaling pathways (92, 93). The activated of the PI3K/Akt has been demonstrated to vascular formation and remodeling. Li et al. proved that the PI3K/AKT pathway can facilitate the migratory and reproductive actions of endothelial progenitor cells by activating Sirt1 (93). Moreover, MiR-217 is abundantly expressed in the plasma of individuals with atherosclerosis. Reducing the expression of miR-217 can ameliorate the development of atherosclerosis by specifically targeting sirtuin 1 and preventing macrophage cell death and inflammatory response. Furthermore, miR-217 has the ability to suppress the apoptosis of ECs (94).

Yao et al. treated H9c2 cells with hypoxic conditions and evaluated migration, proliferation, apoptosis, and invasion. The connection between miR-217 and both Malat1 and Sirt1 was investigated through a dual luciferase reporter experiment and qRT-PCR analysis. This research demonstrated that exposing H9c2 cells to low oxygen levels results in harm, as it limits their ability to grow, move, and spread, and instead triggers programmed cell death. The presence of hypoxia resulted in a significant rise in Malat1 levels. The RNA molecule Malat1 established a robust relationship with miR-217, subsequently resulting in the discovery of Sirt1 as a targeted entity for miR-217. Enhancing Malat1 suppression further worsened the negative effects of hypoxia on H9c2 cells, as miR-217 levels were heightened. However, promoting Sirt1 expression alleviated H9c2 cell damage by activating the PI3K/AKT and Notch pathways. Their reported data indicate that Malat1 has a significant role in causing damage to heart muscle cells under low oxygen conditions via controlling the miR-217-mediated Sirt1 and subsequent signaling pathways: Notch and PI3K/AKT (29).

The ability of miR-558 to selectively attach to and inhibit the creation of MyD88, a protein responsible for activating the NF-κB pathway, a signaling process connected to inflammation and cell survival. Through the inhibition of MyD88, miR-558 can diminish the inflammatory response and facilitate the programmed cell death of cardiac cells (95). In addition, miR-558 has the ability to attach to and inhibit the synthesis of ULK1, leading to the hindrance of autophagy and the increase of cancer cell death through apoptosis (96, 97). In this line, ULK1 has the ability to control the function of mitochondria, which holds a crucial role in regulating apoptosis. ULK1 has also the ability to trigger the division of mitochondria into smaller fragments, a process known as mitochondrial fission. Additionally, ULK1 may initiate the breakdown of damaged mitochondria, called mitophagy. Through its actions, ULK1 can stop cytochrome c from releasing, which would otherwise set off the caspase cascade and cause apoptosis (98, 99).

Disrupting the expression of MALAT1 led to a considerable reduction in cellular survival and an elevated occurrence of cell death in H9C2 cells exposed to isoproterenol (ISO). Furthermore, they conducted a thorough examination of the potential target and determined that miR-558 is directly targeted by MALAT1, as confirmed by the dual luciferase reporter test. They suggested that MALAT1 acts as a decoy, effectively absorbing miR-558. Cells which were transfected by miR-558 mimic showed higher apoptosis. Their findings also showed that miR-558 inhibited ISO-induced protective autophagy and downregulated ULK1 expression. In the end, they utilized a mouse model with knocked-out MALAT1 to confirm that MALAT1 serves as a protective agent for cardiomyocytes against apoptosis and slightly improves cardiac function after ISO treatment. Therefore, they recommended that targeting MALAT1 could potentially be a technique to protect cardiomyocytes during MI (33).

When the body experiences OGD and oxidative stress, ERK is capable of triggering cell death by altering the MFN 1 through phosphorylation. This change causes the MFN1 to bind more tightly to Bak, a protein from the Bcl-2 family that is responsible for triggering programmed cell death (100). Triggering a MAPK signaling pathway can result in both promoting cell survival and inducing cell death through apoptosis. One potential solution to understanding this paradox is to thoroughly examine the key aspects of MAPK signaling networks. The heightened sensitivity of MAPKs and the presence of activation thresholds can help clarify why extended or vigorous stimulation of MAPKs results in cellular demise, whereas short-lived or moderate stimulation provides a protective effect against cell death (101).

Fan et al. created Sprague-Dawley (SD) rat model of MI. In the MALAT1 group (*n* = 10), the pcDNA-MALAT1 plasmids upregulated lncRNA MALAT1, while in the siMALAT1 group, its expression was suppressed. An additional group, referred to as the Sham group, was established with a sample size of 10. They disclosed that the lnc-MALAT1 levels were markedly elevated in the MALAT1 group, while being significantly reduced in the siMALAT1 group (*p* < 0.05), showing successful transfection. The experimental group, in which MALAT1 was inhibited, demonstrated a notable improvement in ejection fraction and left ventricular function (with a significance level of *p* < 0.05). This suggests that blocking MALAT1 has the ability to increase cardiac function after an acute MI. The findings of both the HE staining and TUNEL assay demonstrated that the group administered with siMALAT1 displayed a reduced level of damage in cardiac function and a decline in cell death, in contrast to the group treated with MALAT1. In contrast, the mRNA levels of Collagen I and III, ERK2, Caspase3, and MAPK were considerably soared in the MALAT1 group, whereas the mRNA level of Bcl-2 was notably diminished (*p* < 0.05). The previously mentioned phrases displayed opposite behaviors within the siMALAT1 group. Furthermore, there was a significant elevation in the levels of ERK2 and MAPK protein expressions within the MALAT1 group (*p* < 0.05). Their results demonstrate that the ERK/MAPK pathway is suppressed as a result of reduced levels of lncRNA MALAT1 in Sprague-Dawley rats, leading to a significant improvement in heart function after MI (32).

### 2.3 MALAT1 and proliferation in MI

Mice, in contrast to other animals, are unable to heal from cardiac injury because they quickly lose their capability to promote injury-related CM proliferation shortly after birth (102). Humans also have a similar difficulty to promote the growth of CMs following injury, particularly after the age of 20 (103). Nonetheless, several articles in recent years have demonstrated that it may be feasible to induce CM proliferation and facilitate functional regeneration following myocardial damage. Researchers have examined the involvement of macrophages and monocytes in the proliferative and inflammatory stages of cardiac repair following MI (104). These immune cells can impact the development of cardiomyocytes by releasing growth factors, cytokines, and ECM components (104). In addition, MALAT1 has been identified as a regulator of various pathological processes, comprising proliferation, autophagy, apoptosis, and pyroptosis in ECs (ECs) or cardiomyocytes (15, 105). MALAT1 expression was increased under hypoxic conditions, which facilitated the proliferation and migratory capabilities of cardiac stem and progenitor cells by sponging miR-125 or miR-155, respectively (41, 105).

TGF-β is an important proliferative pathway in many situations including inflammation, fibrosis, and cancer (106). The activation of TGF-β begins with ligand release. The ligands are produced in numerous body cells, not just the myocardium. Before binding to target receptors, they are secreted inactive and need proteolytic cleavage (107). Different types of cells, such as fibroblasts and smooth muscle cells, have the ability to increase in number simply by producing these activating proteins. However, for certain cell types like ECs, the presence of TGF-β can actually hinder the process of cell proliferation. The extent of this influence may depend on the concentration of the TGF-β growth factor (108). Although TGF-β ligands are not unique to the myocardium and are expressed in various cell types, they undergo significant alterations in expression following myocardial damage (109).

Huang et al. developed a MI model through artificial closure of the coronary artery in mice. Protein expression level was analyzed by means of Western blot analysis, whereas RNA expression was calculated using RT-qPCR. Echocardiography was employed to access cardiac function. Masson's trichrome staining allowed for the visualization of the fibrotic area in hearts affected by MI. In both heart infarctions and cardiac fibroblasts treated with angiotensin II (AngII), the expression of MALAT1 was determined higher, while the expression of miR-145 was reduced. Furthermore, the decrease in miR-145 levels was restored upon depletion of MALAT1. Silencing the MALAT1 gene resulted in enhanced cardiac function and suppressed fibroblast growth, collagen production, and *α*-SMA levels induced by AngII in cardiac cells. MiR-145 had a vital function in controlling the expression levels of MALAT1 and manipulating the functioning of TGF-β1. Inhibiting the expression of MALAT1 effectively blocked the stimulation of TGF-β1 caused by AngII in heart fibroblasts. The results of their research suggested that MALAT1 increases the severity of heart fibrosis and negatively impacts the functioning of the heart after a heart attack. This is achieved by regulating the TGF-β1 activity through the presence of miR-145 (31).

MiR-125 specifically bind to and prevent the expression of p53, a gene that involves in suppressing tumor growth by triggering programmed cell death and interrupting the cell cycle. Moreover, miR-125 can shield cardiomyocytes against ischemia-reperfusion damage by suppressing p53 activity (110). MiR-125 binds to and block the activity of JMJD6, leading to the suppression of the producing pro-inflammatory and pro-apoptotic genes, comprising TNF-α, IL-6, and Bax (111). JMJD6 has the ability to control the production of HIF-1*α*, which induces angiogenic genes expression in limited oxygen environments (112). Additionally, JMJD6 can also modulate the expression of miR-21, a microRNA that exhibits an opposite pattern to miR-125. MiR-21 has the capacity to specifically bind to and limit the activity of PTEN, a gene that suppresses the PI3K/AKT pathway. This system involves in regulating cell survival, proliferation, and the angiogenesis. Through the inhibition of PTEN, miR-21 has the ability to suppress apoptosis and inflammation in cardiomyocytes (113).

Li et al. showed that the use of CoCl2 to induce hypoxia can significantly increase the growth and movement capabilities of cardiac progenitor cells (CPCs). Furthermore, the hypoxia-induced CPCs model treated with CoCl2 showed a remarkable increase in the level of MALAT1 expression. When exposed to limited oxygen levels, the cessation of MALAT1 function hindered the reproduction and movement of CPCs. Furthermore, MALAT1 functioned as a reservoir for miR-125. Once miR-125 was suppressed and MALAT1 levels were decreased in a low oxygen environment, the ability of CPCs to grow and move was restored. The expression of JMJD6 was directly affected by miR-125, as miR-125 was responsible for suppressing its levels. Inhibiting the expression of JMJD6 led to the prevention of the protective effect of the miR-125 inhibitor on CPC activity when exposed to hypoxic conditions. Overall, their research demonstrated that MALAT1 is capable of controlling the growth and movement potential of CPCs by means of the miR-125/JMJD6 pathway in hypoxic environments (41).

## 3 MALAT1 and prognosis of MI

In addition to the potentials of lncRNAs in prevention and treatment of MI, which can be performed through modulating the downstream miRNAs expression (114), They can serve as indicators for the early detection and prognosis of MI by identifying the intensity and scope of heart muscle injury, the level of cardiac impairment, and the development of sequelae (115, 116). The objective is to assess the predictive value of the MALAT1 genetic variation in major adverse cardiac and cerebrovascular events (MACCEs). Zhang et al. have conducted a study on 617 individuals with MI and 1,125 control participants. The MALAT1 rs3200401 genotype was detected using SNPscan™ typing tests. The research utilized graphical plots and statistical analysis to examine the connection between variations in the MALAT1 gene and the occurrence of major adverse cardiac and cerebrovascular events (MACCEs). They found significantly higher frequencies of CT + TT and T allele in MACCES. Considering the patients with MI compared to the total population of the study, the T allele frequencies were 15.3% vs. 19.5% (*P* = 0.047) and 14.1% vs. 20.7% (*P* = 0.014), respectively. On the other hand, The CT + TT genotypes frequencies were 28.1% vs. 37.4% (*P* = 0.013) and 25.8% vs. 39.5% (*P* = 0.003), respectively. Nevertheless, among the control participants, there was no significant increase in the frequencies of the T allele (*P* = 0.860) or CT + TT genotypes (*P* = 0.760) in individuals with MACCEs compared to those without MACCEs. Furthermore, the Kaplan-Meier curve examination indicated that individuals possessing the rs3200401 CT + TT genetic variations, both in the overall group and in individuals with MI, had a higher incidence of MACCEs in comparison to those with the CC genotype (with a significance of *P* = 0.015 and *P* = 0.001, respectively). Nonetheless, the control subjects did not show comparable outcomes (*P* = 0.790). The multivariate Cox regression analysis results indicated that individuals with the CT + TT genotypes had a 1.554 times greater chance of experiencing major adverse cardiac and cerebrovascular events (MACCE) than those with the CC genotype. This research also showed that the presence of the CT + TT genotypes of the MALAT1 rs3200401 gene may elevate the risk of MACCEs in patients with MI. This indicates that the MALAT1 gene has the ability to be used in predicting a negative prognosis in MI patients (25).

In another research, Li et al. examined the association between MALAT1 and the risk, characteristics, cytokines, and prognosis of patients with AMI. They recruited a total of 160 patients who were recently determined to have AMI, as well as 50 control individuals who had angina pectoris. Peripheral blood mononuclear cells (PBMCs) were collected in order to quantify MALAT1 using RT-qPCR. The presence of serum cytokines in AMI patients was determined using ELISA. Furthermore, patients with AMI were monitored to assess their risk of experiencing major adverse cardiovascular events (MACE). They demonstrated elevated expression of Lnc-MALAT1 in patients with AMI compared to the control group (median: 2.245 vs. 0.996, *p* = 0.004). Additionally, MALAT1 strongly distinguished between AMI patients and controls, as indicated by an area under the curve of 0.823. Moreover, MALAT1 had a significant positive correlation with LDL cholesterol, CRP, cardiac troponin I, and infarct size in patients with AMI. However, there was no significant association observed with other biochemical markers. Concurrently, the results indicate that the MALAT1 is closely associated with high levels of inflammatory cytokines TNF-alpha, IL-17A, and IL-6, as well as intercellular adhesion molecule-1 and vascular cell adhesion molecule-1, in individuals diagnosed with AMI. Significantly, following categorization, there was a considerable association between high levels of MALAT1 (compared to the low levels) and an increased rate of MACE accumulation (*p* < 0.05). Concluding from this study, MALAT1 can be used for assessing the risk of AMI, determining the severity of the infarct, evaluating inflammatory levels, and predicting prognosis (37). MALAT1 sponges miR-126, miR-155, and miR-30e. Plasma level of miR-30e serves as an indicator for the likelihood of experiencing no-reflow during primary PCI, while miR-126 could potentially serve as a predictive indicator for coronary slow flow. In this regard, Yang et al. assessed the ability of the mentioned genes to accurately predict the likelihood of experiencing the no-reflow phenomenon in patients with ST-segment elevation MI (STEMI) who have undergone primary percutaneous coronary intervention (PCI). The performance of ROC analysis revealed that the quantities of MALAT1, miR-126, miR-30e, and CRP have the potential to function as indicators for discriminating between the outcomes of normal reflow and no-reflow in individuals who have undergone primary PCI. According to the research findings, MALAT1 plays a role in suppressing the production of three miRNAs – miR-30e, miR-126, and miR-155. Furthermore, miR-126 and miR-155 have a specific effect on inhibiting the expression of their target genes, HPSE and EDN1 respectively. Results also showed that miR-30e, miR-126, and miR-155 directly interact with and negatively regulate the activity of CRP, HPSE, and EDN1. Besides, MALAT1 has the potential to serve as a reliable biomarker for the no-reflow phenomena in patients with STEMI who have primary PCI (26).

## 4 MALAT1 as diagnostic biomarker for MI

Cardiotropin I (cTnI) and troponin T (cTnT) are the most preferred diagnostic biomarkers for AMI at the present time (117). Circulating cTnI and cTnT have been widely regarded as the most reliable method for diagnosing AMI for more than two decades. This is because they can be found in the bloodstream within 2–4 h post AMI (118, 119). The amount of circulating cTnI and cTnT show their highest levels 24–48 h after an AMI and remain elevated for even more than a week (120). Recently, high-sensitive cTnI and cTnT have been created to enhance the sensitivity and precision of diagnosing (121, 122). Nevertheless, there are instances where individuals with chronic kidney illnesses, heart failure, and sepsis, particularly older patients, may experience false positive results with increased cTn levels (123–126). Furthermore, due to the prolonged presence of cTn in the bloodstream for more than 7 days, it is doubtful that tiny repeated heart attacks occurring after a massive heart attack may be detected. Thus, it is crucial to find accurate biomarkers that can diagnose STEMI at a very early stage, as well as particular biomarkers that can track the complete disease progression of AMI. Researchers in the United Kingdom (127), the United States (128), and China (129) came to the same conclusion in 2008: circulating miRNAs are sensitive cancer and illness biomarkers. Groundbreaking studies showed that miRNAs have significant promise as biomarkers for AMI (130, 131).

Wang et al. evaluated the diagnostic potentials of lncRNAs obtained from PBMCs. They studied the expression levels of 10 distinct lncRNAs known to be connected with cardiovascular disease in PBMCs taken from 132 AMI patients and 104 unaffected individuals. Employing quantitative RT-PCR analysis, the researchers collected blood samples from the AMI patients after their heart attack. Results showed heightened levels of lncRNAs H19, MALAT1, and MIAT in the AMI patients compared to the control group, among the 10 lncRNAs studied. The ROC analysis demonstrated that peripheral blood mononuclear cell-derived H19 had substantial diagnostic utility for acute MI (ROC, 0.753; 95% confidence interval, 0.689–0.817). Their results also showed that the AUC of MALAT1, as an indicator for diagnosis, was 0.636 (95%CI: 0.561˜0.712) with a cut-off point of 1.79, specificity of 0.75, and sensitivity of 0.555. Therefore, the potentials of the increased levels of H19, MIAT, and MALAT1 generated from PBMCs should be regarded as diagnostic biomarkers for acute MI (35). In another study on 160 patients who were recently diagnosed with AMI and 50 control patients with angina pectoris, Li et al. demonstrated that MALAT1 expression level was substantially elevated in AMI group, indicating the MALAT1 ability to distinguish between AMI and control individuals. In this regard, the AUC of MALAT1 was 0.823. In addition, its level was straightforwardly correlated with infarct size, cardiac troponin I, LDL cholesterol, and CRP in a positive way. Following categorization, there was a notable association between high levels of lnc-MALAT1 (compared to low levels) and an increased rate of MACE accumulation (*p* = 0.035). These findings indicate that Lnc-MALAT1 has the potential to be utilized in evaluating the susceptibility to AMI as a biomarker, determine the extent of the infarct, evaluate inflammatory levels, and predict prognosis (37).

Barbalata et al. examined the potentials of plasma MALAT1 to differentiate between individuals with unstable CAD and those with stable CAD. A number of 23 individuals with stable angina (SA), 21 individuals with unstable angina (UA), and 50 patients with STEMI were included in the study. ROC analysis demonstrated a positive correlation between elevated levels MALAT1 with the presence of UA. In addition, MALAT1 was also correlated with the occurrence of MACE in patients with STEMI. The accuracy of this forecast was improved by incorporating the degree of miR-142-3p in the comprehensive statistical model. They suggested MALAT1 as a valuable diagnostic indicator for identifying vulnerable CAD (40).

## 5 Conclusion

Recent pieces of evidence have revealed that MALAT1 holds a substantial significance in the growth, progress and management of cardiovascular diseases, especially in cases of myocardial ischemia and reperfusion damage. According to research, MALAT1 is implicated in processes such as autophagy, apoptosis, cell proliferation, and inflammation in the cardiovascular system. In investigations involving postconditioning models and acute MI, for example, there was an increase in MALAT1 expression, showing a direct relationship with disease severity. The research performed on individuals with ST-elevation MI validates this finding, as it has shown that higher levels of MALAT1 can be indicative of significant negative outcomes related to the heart. Interestingly, interventions, such as lowering MALAT1 levels *in vivo* and *in vitro*, demonstrated anti-injury effects by suppressing cell death and boosting cell survival. This evidence emphasizes the potentiality of targeting MALAT1 as a therapeutic strategy. Furthermore, the association of MALAT1 with miRNAs such as miR 155 5p, miR 142 3p, and miR 30e emphasizes its role in gene expression relevant to cardiovascular diseases. These interactions have an effect on processes such as collagen formation, inflammation, and cell damage. This underscores the significance of MALAT1 in these situations. To conclusion, MALAT1's diverse role in myocardial ischemia and damage disorders places it not only as an important biomarker for disease development and prognosis, but also as a viable target for therapeutic approaches.

Currently, the intricate three-dimensional structure of lncRNAs, which allows them to interact with various RNA and protein partners, as well as their multifaceted mode of action, have hindered the acquisition of knowledge on lncRNA functions in both normal and pathological situations. However, cutting-edge biochemical methods for studying lncRNAs are continuously being developed, which may uncover previously unknown functions of these molecules, including MALAT1. This could shed more light on their diverse biological activities and their precise role in disease pathobiology. Moreover, significant progress has been made in finding therapeutic agents to target oncogenic lncRNAs in cancer cells, and researchers are now exploring new avenues to design and produce small molecules that can bind to lncRNAs in their three-dimensional conformations. Through targeted genetic deletion of MALAT1 using zinc finger nucleases, as well as MALAT1 therapeutic targeting using synthetic oligonucleotides such as siRNAs and the newly developed LNA gapmeR ASOs, the oncogenic role of this lncRNA and its potential as a target for therapeutic purposes has been established. While further studies utilizing advanced and physiologically relevant *in vivo* models that replicate certain types of cancer are necessary, the current evidence suggests that MALAT1 may hold promise as a primary candidate for future clinically applicable lncRNA-based therapies against MI.
